# Selectivity and Maximum Response of Vibegron and Mirabegron for β_3_-Adrenergic Receptors

**DOI:** 10.1016/j.curtheres.2022.100674

**Published:** 2022-05-14

**Authors:** Benjamin M. Brucker, Jennifer King, Paul N. Mudd, Kimberly McHale

**Affiliations:** 1 Departments of Urology and Obstetrics and Gynecology, NYU Langone Health, New York, New York; 2 Urovant Sciences, Irvine, California; 3 Currently at: Cyclerion Therapeutics, Boston, Massachusetts; 4 Currently at: Priovant Therapeutics, Durham, North Carolina; 5 Dermavant Sciences, Morrisville, North Carolina

**Keywords:** adrenergic receptor, β-adrenoreceptor, overactive bladder, pharmacology

## Abstract

**Background:**

The β_3_-adrenergic agonists vibegron and mirabegron have shown favorable safety profiles and efficacy for the treatment of overactive bladder. However, β-adrenergic receptors are also found outside the bladder, which could lead to off-target activity.

**Objective:**

This study assessed the selectivity of vibegron and mirabegron for β-adrenergic receptors and the maximal effect and potency for β_3_-adrenergic receptors.

**Methods:**

Functional cellular assays were performed using Chinese hamster ovary-K1 cells expressing β_1_-, Chinese hamster ovary cells expressing β_2_-, and human embryonic kidney 293 cells expressing β_3_-adrenergic receptors. Cells were incubated with vibegron, mirabegron, or control (β_1_ and β_3_, isoproterenol; β_2_, procaterol). Responses were quantified using homogeneous time-resolved fluorescence of cyclic adenosine monophosphate and were normalized to the respective control. Half-maximal effective concentration and maximum response values were determined by nonlinear least-squares regression analysis.

**Results:**

Activation of β_3_-adrenergic receptors with vibegron or mirabegron resulted in concentration-dependent β_3_-adrenergic receptor responses. Mean (SEM) half-maximal effective concentration values at β_3_-adrenergic receptors were 2.13 (0.25) nM for vibegron and 10.0 (0.56) nM for mirabegron. At a concentration of 10 µM, β_3_-adrenergic activity relative to isoproterenol was 104% for vibegron and 88% for mirabegron. Maximum response at β_3_-adrenergic receptors was 99.2% for vibegron and 80.4% for mirabegron. β_1_-adrenergic activity was 0% and 3% for vibegron and mirabegron, respectively; β_2_-adrenergic activity was 2% and 15%, respectively.

**Conclusions:**

Vibegron showed no measurable β_1_ and low β_2_ activity compared with mirabegron, which showed low β_1_ and some β_2_ activity. Both showed considerable selectivity at β_3_-adrenergic receptors; however, vibegron demonstrated near-exclusive β_3_ activity and a higher maximum β_3_ response.

## Introduction

Overactive bladder (OAB) is highly prevalent in adults^1^ and is characterized by symptoms such as urgency with or without urge urinary incontinence.^2^^,^^3^ First-line treatment for OAB includes behavioral therapy with or without pharmacotherapy; second-line treatment includes oral anticholinergics and β_3_-adrenergic receptor agonists.^2^^,^^4^ However, treatment with anticholinergic agents is associated with bothersome side effects such as dry mouth and constipation^5^ that can limit treatment persistence, as well as an increased risk of falls and potential for impaired cognitive function.^6^, ^7^, ^8^ The β_3_-adrenergic receptor agonists are a class of treatment for OAB that minimize several of the adverse effects associated with anticholinergic use.^9^^,^^10^ Vibegron and mirabegron are β_3_-adrenergic receptor agonists that are currently approved in the United States, Europe (mirabegron only), and Japan for the treatment of OAB.^11^^,^^12^

The β-adrenergic receptors are G protein-coupled receptors that vary in structure, expression, and function. The human β_3_-adrenergic receptor shares approximately 50% of its sequence with the β_1_- and β_2_-receptors.^13^^,^^14^ Compared with β_2_-, the β_3_-adrenergic receptor lacks C-terminal phosphorylation sites that in β_2_-adrenergic receptors are associated with agonist-induced desensitization.^15^ In the bladder and detrusor muscles, β_3_-adrenergic receptors account for 94% to 97% of β-adrenergic receptor mRNA.^16^^,^^17^ The primary function of β_3_-adrenergic receptors in the bladder is to aid in detrusor smooth muscle relaxation during the filling stage of the micturition cycle.^17^^,^^18^ In addition to the bladder and detrusor muscle, β-adrenergic receptors are expressed on cardiovascular tissue (reviewed in Wachter et al^19^), inviting concerns about potential off-target effects associated with the use of β-adrenergic agonists if they are not highly selective for a given receptor subtype. Beyond selectivity, potency at β_3_-adrenergic receptors may influence efficacy.

Both vibegron and mirabegron act on the β_3_-adrenergic receptor; however, there are innate differences associated with their unique pharmacologies. Because earlier generations of β_3_-adrenergic agonists were associated with the buildup of toxic metabolites and with off-target effects, the structure of vibegron was carefully chosen and intentionally designed to improve upon β_3_-adrenergic agonists that had failed preclinically.^20^^,^^21^ Mirabegron has been shown to stimulate β_1_-adrenergic receptors at supratherapeutic doses, leading to increases in contractile force in the atrium.^11^^,^^22^

Additional differences between vibegron and mirabegron include dose and titration requirements. In the Phase III EMPOWUR and EMPOWUR extension studies, once-daily vibegron 75 mg showed safety and efficacy for the treatment of OAB^10^^,^^23^ at a single dose strength. Steady state concentrations of vibegron are reached within 7 days of once-daily dosing, and the effective half-life is 30.8 hours. For the treatment of OAB, the recommended 25-mg starting dose of mirabegron has an onset of action of up to 8 weeks,^10^ and therefore dose escalation to 50 mg may be required. Mirabegron 50 mg has been shown to be effective within 4 weeks.^11^^,^^24^

The selectivity of vibegron and mirabegron for β-adrenergic receptors has not been tested in a head-to-head fashion. The aim of this study was to assess and compare the selectivity of vibegron and mirabegron for each β-adrenergic receptor subtype, as well as the maximal effect and potency for β_3_-adrenergic receptors.

## Methods

### Cells and cell culture

Chinese hamster ovary (CHO)-K1, CHO, and human embryonic kidney (HEK) 293 cells stably expressing human β_1_-, β_2_-, or β_3_-adrenergic receptors, respectively, and HEK293 and CHO-K1 cells expressing human α_1D_- and α_2B_-adrenergic receptors, respectively, were provided by Eurofins Panlabs (Taipei, Taiwan). Cell lines were cultured at 37°C with 5% carbon dioxide. CHO-K1 and CHO cells were cultured in Dulbecco's modified Eagle's medium/Ham's-F12 supplemented with 2 mM l-glutamine and 10% (v/v) heat-inactivated fetal calf serum. HEK293 cells were cultured in Eagle's minimal essential medium and Earle's balanced salt solution supplemented with 1% (v/v) minimal essential medium nonessential amino acids, 2 mM l-glutamine, and 10% (v/v) heat-inactivated fetal calf serum.

### Selectivity and potency assays

Cells were plated at a density of 2.5 × 10^5^ cells/mL. For β-receptor selectivity and potency, assays were performed in an incubation buffer consisting of Hank's balanced salt solution containing 5 mM HEPES, 0.1% (w/v) bovine serum albumin, and 100 µM 3-isobutyl-1-methylxanthine (a phosphodiesterase inhibitor), pH 7.4. For α-receptor selectivity, assays were performed in an incubation buffer consisting of 50 mM Tris·HCl, pH 7.4; for α_2B_ assays, incubation buffer was supplemented with 1 mM EDTA, 12.5 mM magnesium chloride, and 0.2% (w/v) bovine serum albumin. Test compounds for β-receptor assays were diluted in 0.4% (v/v) dimethyl sulfoxide (DMSO) and for α-receptor assays were diluted in 1.0% (v/v) DMSO. For β_1_-, β_2_-, α_1D_-, and α_2B_-adrenergic receptor activity, compounds were tested at a single concentration (10 µM). For β_3_-adrenergic receptors, compounds were serially diluted in DMSO and aliquoted into 96-well microtiter plates in assay buffer with IBMX. Reagents were purchased from MilliporeSigma (Burlington, Massachusetts) and CisBio (Bedford, Massachusetts).

For β-receptor assays, cyclic adenosine monophosphate (cAMP) accumulation was assessed by a time-resolved fluorescence resonance energy transfer (TR-FRET) immunoassay (CisBio cAMP Dynamic) following manufacturer's instructions. CHO-K1 cells expressing β_1_-adrenergic receptors were incubated for 15 minutes at 37°C with vibegron, mirabegron, or control (isoproterenol); CHO cells expressing β_2_-adrenergic receptors and HEK293 cells expressing β_3_-adrenergic receptors were incubated for 20 minutes at 37°C with vibegron, mirabegron, or control (procaterol for β_2_, isoproterenol for β_3_). After incubation, cells were lysed by the addition of a detection buffer containing a europium-labeled cAMP tracer. Fluorescence was measured 1 hour following incubation at room temperature (excitation, 320 nm; emission, 620 and 665 nm). For each assay, a cAMP standard curve was used to convert fluorescence readings to cAMP levels. Assays for specificity were performed using 2 biological replicates for β_1_ and β_2_ and 3 biological replicates for β_3_. Doses used to obtain half-maximal effective concentration (EC_50_) ranged from 0.3 nM to 10 µM.

To test selectivity of vibegron and mirabegron to β-adrenergic receptors, inhibition of α_1D_ and α_2B_ was also assessed. HEK293 and CHO-K1 cells expressing α_1D_ and α_2B_-adrenergic receptors, respectively, were incubated with radioligand (0.60 nM [^3^H]-prazosin for α_1D_ or 2.50 nM [^3^H]-rauwolscine for α_2B_) and vibegron, mirabegron, or control inhibitor (0.88 nM prazosin for α_1D_ or 14 nM yohimbine for α_2B_) for 60 minutes at 25°C. Membranes were filtered and washed 3 times, and the filters were counted with a scintillation counter to determine binding. These radioligand binding assays were performed using 2 biological replicates for α_1D_ and α_2B_. Data are presented as the percent inhibition of control radioligands; criterion for significance was ≥50% stimulation or inhibition.

### Statistical analysis

Significance criteria for the agonists (ie, vibegron and mirabegron at each β receptor) was considered a >0% increase in cAMP relative to isoproterenol or procaterol. Percent activity was defined as the maximal response of the test compound concentration expressed as percentage of the maximal response to the full control agonist (isoproterenol for β_1_ and β_3_ or procaterol for β_2_). EC_50_ and maximum response (E_max_) values were determined by nonlinear least squares regression analysis. A gamma coefficient was calculated to determine the measure of association between E_max_ and EC_50_. Comparison of activity was made between vibegron and mirabegron at each β receptor.

## Results

### β-Adrenergic receptor specificity

β_1_-adrenergic activity relative to isoproterenol was 0% and 3% for vibegron 10 µM and mirabegron 10 µM, respectively; β_2_-adrenergic activity relative to procaterol was 2% and 15%. At a concentration of 10 µM, which exceeds mean human C_max_ values of vibegron and mirabegron by >10 times, β_3_-adrenergic activity relative to isoproterenol was 104% for vibegron and 88% for mirabegron. Neither vibegron nor mirabegron met the significance criterion for inhibition of α_1D_- or α_2B_-adrenergic receptors. α_1D_-adrenergic activity relative to prazosin was 3% and 20% for vibegron and mirabegron, respectively; α_2B_-adrenergic activity relative to yohimbine was 37% and 33%_._

### β_3_-Adrenergic receptor maximal effect and potency

**Figure 1:**
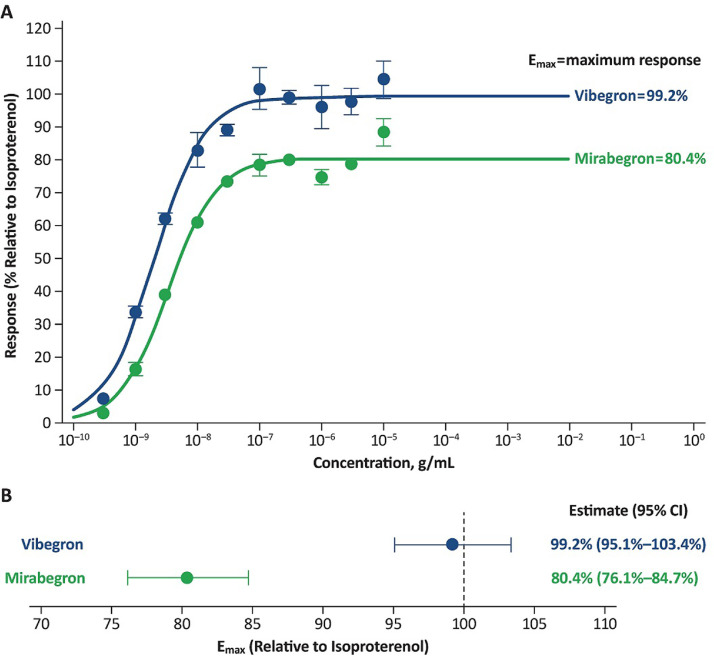
(A) Concentration-response curves (mean [SEM]) for vibegron and mirabegron at β_3_-adrenergic receptors relative to the full agonist (isoproterenol [control]). (B) Mean (95% CI) maximum response (E_max_) for vibegron and mirabegron at β_3_-adrenergic receptors relative to the full agonist (isoproterenol [control]).

**Figure 2:**
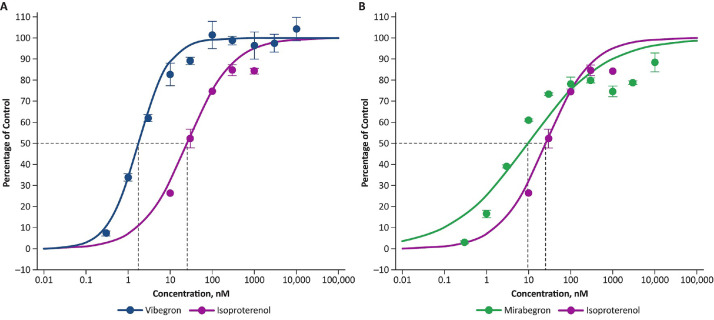
Concentration-response curves (mean [SEM]) for (A) vibegron and (B) mirabegron versus isoproterenol (control) at β_3_-adrenergic receptors. Dashed lines indicate EC_50_ values.

The E_max_ for vibegron and mirabegron at the β_3_-adrenergic receptor was estimated to be 99.2% and 80.4%, respectively, relative to isoproterenol (Figure 1 and Table 1). Treatment of β_3_-adrenergic receptor‒expressing HEK293 cells with vibegron, mirabegron, or isoproterenol resulted in concentration-dependent responses at β_3_-adrenergic receptors (Figure 2). The mean (SEM) EC_50_ values at the β_3_-adrenergic receptor were 2.13 (0.25) nM for vibegron and 10.0 (0.56) nM for mirabegron.

**Table 1:** Parameter estimates for vibegron and mirabegron at β_3_-adrenergic receptors

Parameter	Vibegron	Mirabegron
E_max_, %*		
Mean (SE)	99.2 (1.8)	80.4 (1.8)
CV	1.8	2.3
95% CI	95.1‒103.4	76.1‒84.7
EC_50_, nM		
Mean (SE)	2.0 (0.2)	3.4 (0.5)
CV	11.0	13.5
95% CI	1.5‒2.5	2.3‒4.5
Gamma coefficient		
Mean (SE)	1.1 (0.1)	1.1 (0.1)
CV	10.9	13.1
95% CI	0.8‒1.3	0.8‒1.4

CV = coefficient of variation; EC_50_ = half maximal effective concentration; E_max_ = maximum response.

⁎ Relative to the full agonist, isoproterenol (control).

## Discussion

β_3_-adrenergic receptors are highly expressed in bladder tissue and detrusor smooth muscle where they mediate relaxation to aid in bladder filling.^17^^,^^18^ However, the diverse expression patterns of β-adrenergic receptors, including the expression of β_1_ and β_2_ receptors on cardiovascular tissue (reviewed in Wachter et al^19^), complicates the targeting of β-adrenergic receptors. In addition, early drug development programs of β_3_-adrenergic receptor agonists were marked by off-target toxicities associated with drug metabolism and adverse effects such as phospholipidosis,^20^ affirming the importance of the design of β-adrenergic receptor agonists that are highly specific for the β_3_ subtype to avoid off-target effects. This knowledge informed the intentional design of vibegron and aimed to improve drug-like properties and receptor selectivity, as well as reduce adverse effects seen with earlier molecules targeting β-adrenergic receptors.^20^^,^^21^ Such alterations in structure produced a compound that was highly specific and selective toward β_3_-adrenergic receptors.^20^^,^^25^

In this study, direct comparison of vibegron and mirabegron activity showed considerable selectivity of both drugs at β_3_-adrenergic receptors. However, vibegron did not show any measurable β_1_ activity and had low β_2_ activity, whereas mirabegron showed low β_1_ and more β_2_ activity compared with vibegron. These results are in line with prior studies assessing the selectivity and specificity of vibegron or mirabegron at β-adrenergic receptors. In 2 prior studies using transfected CHO cells, mirabegron showed low agonist activity at monkey and human β_1_- and β_2_-adrenergic receptors and high selectivity at β_3_-adrenergic receptors^26^^,^^27^; both studies showed a maximal response of mirabegron relative to isoproterenol at β_3_-adrenergic receptors of 80%. As monkey β_3_-adrenergic receptors have high homology with human β_3_-adrenergic receptors,^26^ these results are congruent with the maximum response of 80.4% seen in this study with mirabegron at human β_3_-adrenergic receptors. Monkey bladder strips under potassium chloride stimulation or resting tension have also shown maximal relaxant effects with mirabegron of 89% and 82%, respectively, relative to papaverine (control)^26^; similar maximal relaxant effects were seen using mirabegron with carbachol-precontracted human or rat bladder tissue (89% and 94%, respectively).^27^ Prior reports in CHO cells transfected with human β_3_-adrenergic receptors have shown that vibegron has a maximum response of 84% at β_3_-adrenergic receptors and minimal activity at β_1_- and β_2_-adrenergic receptors.^20^^,^^25^ In the presence of human serum, however, the maximum response at β_3_-adrenergic receptors was increased to 101% to 102%.^20^^,^^25^ Similarly high maximum responses of 82% to 108% with vibegron were seen for CHO cells transfected with rhesus monkey, rat, or dog β_3_-adrenergic receptors.^20^^,^^25^ Although in vitro activity may not directly translate to clinical significance or reflect pathologic conditions, activity at β_1_ receptors in vitro, for example, could indicate the possibility of in vivo activity on cardiac tissue, where β_1_ is primarily expressed.^19^

Given the expression patterns of β-adrenergic receptors, cardiovascular off-target effects at β_1_- or β_2_-adrenergic receptor agonists are a concern. In the EMPOWUR and EMPOWUR extension studies, adverse events of hypertension were reported in similar percentages of patients who received vibegron and placebo.^10^^,^^23^ In a clinical trial to assess small changes in blood pressure and heart rate using ambulatory blood pressure monitoring, vibegron was not associated with statistically significant or clinically meaningful effects on blood pressure or heart rate in adults with OAB with or without preexisting hypertension.^28^ Although no direct comparison of trials studying mirabegron and vibegron can be made, safety results from these randomized controlled trials, in combination with the high level of selectivity of vibegron at β_3_-adrenergic receptors in this study, suggest that vibegron may be less likely than mirabegron to be associated with off-target effects on the cardiovascular system.

These results are limited by low statistical power because the assays for specificity were performed using 2 biological replicates. Additionally, the receptor density per cell line is unknown. The β-adrenergic receptor activity was assessed using a cAMP assay, although there is evidence of cAMP-independent activity for mirabegron.^29^ Further, as a proof-of-concept study, results seen in vitro may not directly translate to human beings or clinical study and may not reflect pathologic conditions.

## Conclusions

This study enabled direct comparisons of mirabegron and vibegron activation and specificity across the family of β-adrenergic receptors and evaluated the likelihood of off-target effects within this family. Vibegron showed no measurable β_1_ and low β_2_ activity compared with mirabegron, which showed low β_1_ and some β_2_ activity, consistent with previous reports. Both vibegron and mirabegron showed considerable selectivity at β_3_-adrenergic receptors as expected; however, vibegron demonstrated near-exclusive β_3_ activity. Vibegron showed a higher maximum β_3_-adrenergic receptor response, at 99.2% versus 80.4% with mirabegron, consistent with previous reports, and was more potent than mirabegron at activating β_3_-adrenergic receptors. These studies demonstrate high specificity of vibegron to the β_3_-adrenergic receptor and reduced specificity against β_1_ and β_2_ receptors compared with mirabegron.
